# Structure Prediction: New Insights into Decrypting Long Noncoding RNAs

**DOI:** 10.3390/ijms17010132

**Published:** 2016-01-21

**Authors:** Kun Yan, Yasir Arfat, Dijie Li, Fan Zhao, Zhihao Chen, Chong Yin, Yulong Sun, Lifang Hu, Tuanmin Yang, Airong Qian

**Affiliations:** 1 Key Laboratory for Space Bioscience & Biotechnology, Institute of Special Environmental Biophysics, School of Life Sciences, Northwestern Polytechnical University, 127 Youyi Xilu, Xi’an 710072, China; yan@mail.nwpu.cn (K.Y.); Yasir@mail.nwpu.edu.cn (Y.A.); lidijie@mail.nwpu.edu.cn (D.L.); sofan@mail.nwpu.edu.cn (F.Z.); chzhh@mail.nwpu.edu.cn (Z.C.); Yinchong42@gmail.com (C.Y.); yulongsun@nwpu.edu.cn (Y.S.); lifanglinn@mail.nwpu.edu.cn (L.H.); 2 Department of Bone Disease Oncology, Hong-Hui Hospital, Xi’an Jiaotong University College of Medicine, South Door slightly Friendship Road 555, Xi’an 710054, China; doctoryangtm@163.com

**Keywords:** lncRNAs, function, structure prediction, secondary structure, tertiary structure

## Abstract

Long noncoding RNAs (lncRNAs), which form a diverse class of RNAs, remain the least understood type of noncoding RNAs in terms of their nature and identification. Emerging evidence has revealed that a small number of newly discovered lncRNAs perform important and complex biological functions such as dosage compensation, chromatin regulation, genomic imprinting, and nuclear organization. However, understanding the wide range of functions of lncRNAs related to various processes of cellular networks remains a great experimental challenge. Structural versatility is critical for RNAs to perform various functions and provides new insights into probing the functions of lncRNAs. In recent years, the computational method of RNA structure prediction has been developed to analyze the structure of lncRNAs. This novel methodology has provided basic but indispensable information for the rapid, large-scale and in-depth research of lncRNAs. This review focuses on mainstream RNA structure prediction methods at the secondary and tertiary levels to offer an additional approach to investigating the functions of lncRNAs.

## 1. Introduction

The term noncoding RNAs (ncRNAs) refers to RNA transcripts that do not encode proteins [1]. Approximately 93% of human genomic DNA can be transcribed into RNAs [2]. Merely 2% of these RNAs will be translated into approximately 20,000 types of protein translation products, and the remaining 98% of these RNAs represent noncoding RNAs that are rarely translated [3]. ncRNAs can be classified as housekeeping or regulatory ncRNAs. Housekeeping ncRNAs include ribosome RNAs (rRNAs), transfer RNAs (tRNAs), small nuclear RNA (snRNAs) and small nucleolar RNA (snoRNAs), while regulatory RNAs include small interfering RNA (siRNAs), microRNA (miRNAs), long noncoding RNAs (lncRNA), piRNA, natural antisense transcripts (NATs) and circular RNA (circRNAs) [4,5,6,7].

Except for tRNAs and rRNAs, ncRNAs have been traditionally disregarded as “transcriptional noise” [8]. Although proteins have long been considered to carry genetic information, emerging evidence implies that ncRNAs are also involved in the regulation of gene expression that impacts the growth and development of organisms [9,10,11]. Compared with short RNAs (<200 nt), highly transcribed long noncoding RNAs (lncRNAs) (>200 nt) may perform more complex biological functions [12,13,14]. These RNAs have been implicated in the regulation of gene expression at the transcriptional or posttranscriptional level exerting effects on dosage compensation, chromatin regulation, genomic imprinting, nuclear organization, alternative splicing of pre-mRNA and many other biological processes [15,16,17]. Considering the participation of lncRNAs in various aspects of gene expression affecting the differentiation and development of organisms, it is not surprising that the dysregulation of lncRNAs has been involved in disease [18,19]. According to a genome-wide association study, 43% of reported trait/disease-associated SNPs (TASs) were intergenic, suggesting essential roles for ncRNAs in common diseases [20]. Furthermore, Chen *et al.* [21] created lncRNADisease, a database of 166 lncRNA-associated diseases. lncRNADisease collected nearly 480 entries of experimentally validated lncRNA-disease associations. The recognition of the important roles of lncRNAs in human disease has provided novel diagnostic and therapeutic opportunities [22].

Given the wide range of biological functions in which lncRNAs have been implicated, we predict that many more lncRNAs will be determined to have important functions. For many RNAs, there is a close relationship between structure and function [23,24,25]. Their structural diversity allows for RNA to perform various functions, including catalytic, organizational and other regulatory functions [26,27]. Generating structural models of these RNAs that are faithful to their native structures is essential because the structure of RNA influences its transcription, splicing, cellular localization, translation and turnover [28]. Thus, acquiring structural information for RNA is often the first step towards exploring its function [29].

## 2. Review

**Figure 1:**
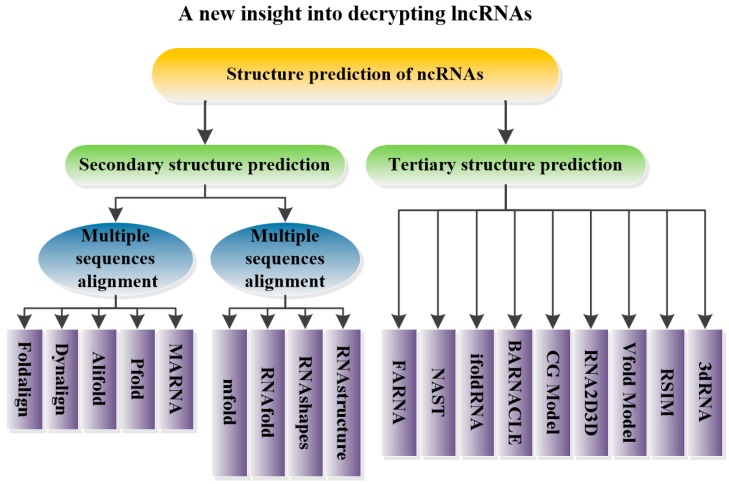
The graphical abstract of this review.

This review focuses on lncRNAs, which comprise the least understood class of ncRNAs. Their functions, mechanisms, roles in epigenetics and relationships with diseases are introduced. Moreover, ncRNA structure prediction methods such as Foldalign [30], Pfold [31], Mfold [32], RNAfold [33], RNAshapes [34], RNAstructure [35], NAST [36], iFoldRNA [37], and 3dRNA [38] are reviewed (Figure 1). Furthermore, the theories underlying each method as well as the advantages and pitfalls of their applications are provided. Based on this summary, another step in the understanding of lncRNAs can be achieved. As the secondary/tertiary structures of several functionally understood lncRNAs have been predicted (or experimentally verified), RNA structure predictions may help identify additional functional lncRNAs and may thus offer clues for the design of targeted small molecule therapeutics to promote drug development and the treatment of diseases [39].

## 3. ncRNAs

Recent advances in the study of ncRNAs have demonstrated the existence of ncRNAs in a wide variety of mammalian transcriptomes. miRNAs, siRNAs, lncRNAs and other ncRNAs have been found to play significant roles in various physiological and developmental processes in eukaryotes, furthering our knowledge of ncRNAs.

### 3.1. Evolutionary Conservation of ncRNAs

A plethora of analyses have revealed that the majority of ncRNAs possess low rates of interspecies conservation at the primary sequence level as protein-coding RNAs [40]. Analyses of the conservation of various classes of ncRNAs have revealed that these RNAs differ in their evolutionary conservation. Of these ncRNA transcripts, miRNAs and snoRNAs are highly sequence-conserved [41,42], even more so than mRNAs or other protein-coding transcripts [43]. Conversely, longer ncRNAs have been found to evolve at a rapid rate, although they possess small conserved segments [44,45]. As opposed to miRNAs, which negatively regulate protein-coding genes by targeting the 3′ UTR (untranlated rigions) of their target mRNAs, longer ncRNAs can negatively or positively regulate protein-coding genes [46,47,48]. Moreover, the known mechanisms underlying the function of lncRNAs are diverse. Considering the dramatic differences in the functions and mechanisms between miRNAs and lncRNAs, distinct evolutionary constraints on these types of RNAs can be easily imagined. Interestingly, a number of researchers have observed the structural conservation of lncRNAs [49]. Some specific structural regions of lncRNAs seem to play regulatory roles, whereas other regions consisting of exact sequences serve only as linkers between different functional modules [50,51,52].

Various technologies have emerged to detect the stability and conservation of ncRNA. Transcriptome sequencing has been applied to the profiling of ncRNAs conservation. First-generation sequencing, proposed by Sanger [53], was based on double deoxidizing chain-termination. The sequencing reads associated with this method can reach 1000 bp with high accuracy; however, due to its high cost and low throughput, it has not been widely used. Compared with first-generation sequencing, next-generation sequencing (NGS) [54] accelerates the sequencing process and exhibits high throughput. Moreover, NGS can be simultaneously utilized for the analysis of ncRNA expression profiling and sequence variation. Whereas the sequencing reads dramatically decrease, polymerase chain reaction (PCR) leads to a high sequencing error rate. Without PCR amplification, single-molecule sequencing (SMS) decreases the error rate associated with NGS. In addition, SMS maintains a high throughput status, a low cost and the ability to produce long sequencing reads [55]. In addition to directly determining the sequences of ncRNAs, there are several databases and types of software to profile the evolutionary conservation of ncRNAs. Basic Local Alignment Search Tool (BLAST) [56], which is based on sequence alignment, is the most widely used program to search for sequence similarity. Other packages such as CLUSTAL X [57], mirTools [58] and MEGA3 [59] have been used for analyzing data derived from NGS to profile the sequence conservation of ncRNAs. Moreover, numerous emerging structure prediction methods offer a new strategy for the study of the structural conservation and function of ncRNAs.

### 3.2. Roles of ncRNAs and the Mechanisms Involved in Their Functions

Many ncRNAs remain undiscovered, and the functions of the majority of previously discovered ncRNAs are not yet known. Furthermore, a low evolutionary conservation of these RNAs has been verified. All of these indications suggested that ncRNAs do not possess biological function. However, mounting evidence suggests that the lack of sequence conservation does not necessarily symbolize a deficiency in function [40]. Increasingly, studies have revealed that ncRNAs are involved in gene expression at almost every level of organismal differentiation and development, impacting processes including transcriptional/post transcriptional regulation, chromatin architecture, translation, alternative splicing of pre-mRNA and many other biological processes [15,16,17,60].

Several mechanisms by which ncRNAs regulate gene expression have been discovered. (1) Piwi-interacting RNAs (piRNAs) have been shown to play roles in the formation of heterochromatin [61]; (2) miRNAs are involved in the modification of histones [62]; (3) various ncRNAs, such as miRNAs and promoter RNAs (pRNAs), play regulatory roles in DNA methylation [63]; (4) some lncRNAs impact the alternative splicing of pre-mRNA [64]; (5) miRNAs degrade mRNAs through binding to the 3′ UTR of their target mRNAs [7]; (6) lncRNAs modulate their structures to recruit specific proteins and form a complex [65]; and (7) interestingly, the newly discovered organellar ncRNAs have been shown to be associated with mitochondria and chloroplasts and represent an emerging mechanism underlying ncRNA-regulated gene expression [66].

### 3.3. ncRNAs in Diseases and Clinical Diagnosis

Because ncRNAs regulate various levels of gene expression and are involved in numerous biological processes, the dysregulation of ncRNAs is linked to diseases. It has been reported that ncRNAs exert significant effects on the immune response, inflammatory lung diseases [67], neurodevelopmental disorders [68] and cancer [69,70,71].

In general, abnormal tissues are obtained by invasive methods for the detection of biomarkers in the diagnosis or clinical treatment of tumors. However, due to the introduction of an external source, this is not the optimal choice for diagnostic and therapeutic applications. The characteristics of stability, specificity, sensitivity, predictability and accessibility are required for quantifiable indicators of diseases [72]. Some ncRNAs have been demonstrated to have potential as biomarkers and therapeutic targets for diseases due to their stabilities and accessibilities without invasive obtainment methods [73]. miRNAs are stable and have been found in biological fluids such as urine, serum, saliva and plasma, allowing miRNAs to be easily detected via non-invasive methods [74,75]. The detection of aberrant expression of miRNAs has been applied to the diagnosis and prognosis of cardiac diseases [76] and autoimmune diseases [77]. A genome-wide analysis has revealed only a fraction of lncRNAs are unstable and surprisingly, intronic, intergenic and cis-antisense lncRNAs are highly stable with a half-life of more than 16 h [78]. Some serum-derived lncRNAs have been used as biomarkers for hepatocellular carcinoma and colorectal cancer with high stability, reproducibility and specificity [79]. Moreover, snoRNAs serve as potential biomarkers for the diagnosis of non-small cell lung cancer (NSCLC) [80] and osteoarthritis progression after anterior cruciate ligament (ACL) injury [81]. Undoubtedly, the understanding of ncRNA function contributes to the development of biomarkers for the prognosis and clinical treatment of diseases.

## 4. Long Noncoding RNAs

**Figure 2:**
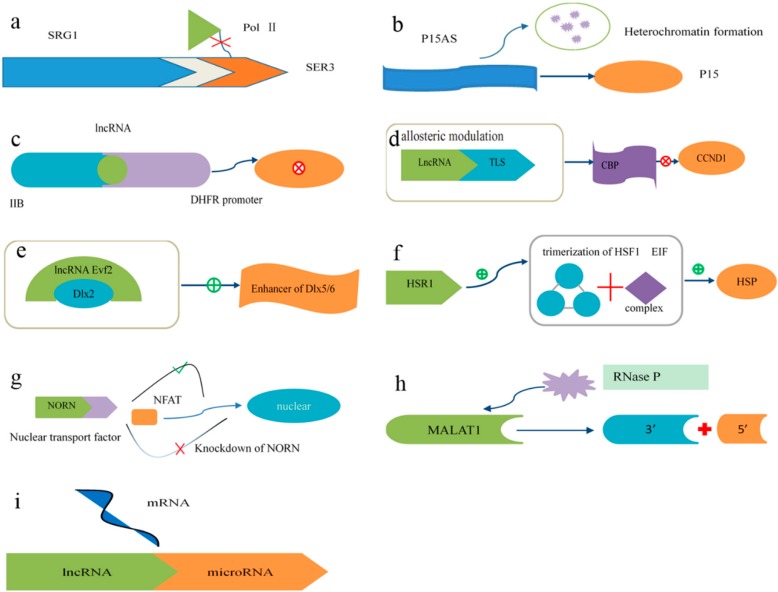
Mechanisms of lncRNA action in transcriptional regulation. (**a**) Transcription of the lncRNA SRG1 inhibits the expression of the SER3 gene by interfering with the binding of RNA polymerase II to DNA; (**b**) The expression of the p15 antisense RNA, the lncRNA of a tumor suppressor gene, results in the silencing of the p15 gene through the induction of heterochromatin formation, which persisted after the p15 antisense RNA was turned off; (**c**) lncRNA binds to the major DHFR promoter and IIB, a general transcriptional factor, to form a stable and specific complex to dissociate the preinitiation complex from the major DHFR promoter; (**d**) As a response to stress, the RNA-binding protein TLS, under allosteric modulation via lncRNA upstream of CCND1, binds to chromatin-binding protein (CBP) and inhibits CBP/P300 HAT activities on CCND1; (**e**) The lncRNA Evf2, a crucial co-enhancer of regulatory proteins involved in transcription, cooperates with the Dlx2 protein to activate the Dlx5/6 enhancer in a target gene; (**f**) In response to heat shock, the lncRNA HSR1 (heat shock RNA-1) promotes the trimerization of HSF1 (heat-shock transcription factor 1), and consequently the translation factor EIF interacts with HSR1 and HSF1 to forms a complex to facilitate the expression of heat-shock protein (HSP); (**g**) NFAT is nuclear factor of activated T cells. The lncRNA NRON (noncoding repressor of NFAT) may form a complex with importin proteins to regulate the subcellular localization of NFAT. The knockdown of NRON increases the expression and activity of NFAT; (**h**) The lncRNA metastasis-associated lung adenocarcinoma transcript 1(MALAT1) has been shown to be abnormally expressed in many human cancers. The nascent MALAT1 transcript is cleaved by RNase P to produce the 3′ end of the mature MALAT1 transcript and the 5′ end of the small RNA; (**i**) Several studies have elucidated that some lncRNAs can act as microRNA sponges to competitively bind to microRNAs and decrease microRNA-induced tumorsphere differentiation.

Long noncoding RNAs (lncRNAs) consist of at least 200 nucleotides [82]. The structural conservation of lncRNAs is stronger than the conservation of their nucleotide sequences. It has been recognized that lncRNA transcription regulates the expression of genes in close genomic proximity in a cis-acting manner [83,84,85,86,87,88] and targets distant transcriptional activators or repressors in a trans-acting manner [89,90]. Additionally, various mechanisms involved in the transcriptional regulation of lncRNAs have been elucidated (some examples are shown in Figure 2) [83,84]. Moreover, lncRNAs also participate in epigenetic gene regulation [91,92]. Models of their functions are shown in Figure 2, where lncRNAs are depicted as playing a variety of roles in cellular networks. Therefore, it is inevitable that the dysregulation of lncRNAs is closely associated with diseases [18,19,93].

### 4.1. Evolutionary Conservation of lncRNAs

Ken C. Pang *et al.* [40] investigated several types of noncoding RNAs that have been demonstrated or predicted to possess functionality, including miRNAs, lncRNAs and snoRNAs. As expected, lncRNAs are less conserved than miRNAs and snoRNAs. However, their findings imply that this lack of conservation does not necessarily dictate a lack of function. Due to the absence of conservation at the nucleotide sequence level, functional studies of lncRNAs are challenging. A number of researchers have uncovered a structural conservation [49]. Some specific structural regions of lncRNAs seem to play regulatory roles, while other regions consisting of exact sequences serve only as linkers between different functional modules [50,51,52].

### 4.2. Mechanisms of lncRNA Function

The known mechanisms involved in the function of lncRNAs are as follows (Figure 2): (a) To induce transcriptional interference, lncRNAs spanning downstream promoter regions of protein-coding genes interfere with transcription factors via binding to their activators and repress the expression of these protein-coding genes [94]; (b) To initiate chromatin remodeling, the transcription of lncRNAs may induce heterochromatin formation and DNA methylation, thus leading to the silencing of tumor suppressor genes [47,95]; (c) lncRNAs bind to basal transcription factors to inactivate their promoters and thus repress the expression of target genes [96]; (d) lncRNAs activate accessory proteins to repress gene expression [83,97]; (e) lncRNAs activate transcription factors to promote the expression of target genes. This reveals a novel mechanism involving the cooperative actions of an lncRNA and a homeodomain protein to regulate transcription [98]; (f) The formation of a trimer containing an activator protein, a translation elongation factor and an lncRNA accelerates the expression of target genes [99]; (g) lncRNAs interact with importin proteins to regulate the subcellular localization of transcription factors. [100]; (h) lncRNAs act as the precursors of small RNAs to perform functions [101]; (i) lncRNAs bind to small RNAs to modulate their activities [102].

### 4.3. Epigenetics

It is reported that lncRNAs participate in the epigenetic regulation of gene expression [103,104,105,106], and recent studies suggests a unified model of their mechanism of action. The lncRNAs may directly or indirectly recruit protein complexes involved in chromosome modifications, which results in epigenetic regulation [91]. In accordance with the relative positional relationship between lncRNAs and their target genes, mechanisms by which lncRNAs regulate target genes can be considered cis [84,85,86,87,88] or trans [83,89]. For those lncRNAs regulating target genes in cis, it was found that the RNAs can form a nuclear complex that is closely linked to the silenced genes. It is suggested that the lncRNAs may bind to epigenetics modifiers to mediate gene silencing [107]. The HOTAIR lncRNA inactivates genes in trans and interacts with Polycomb Repressive Complex 2 (PRC2) to mediate transcriptional silencing of the HOXD locus [90].

### 4.4. LncRNAs and Disease

As mentioned above, an increasing number of studies have demonstrated that lncRNAs participate in th As previously mentioned, an increasing number of studies have demonstrated that lncRNAs participate in the regulation of protein coding genes at the transcriptional and posttranscriptional levels [108]. It is reported that the dysregulation of lncRNAs seems to be the primary cause of many complex human disease processes [109,110], including the development and progression of many types of cancer [111], such as colon cancer [112], prostate cancer [113], breast cancer [114], liver cancer [115], gastrointestinal cancer [116] and other cancers [12,117]. Moreover, some studies have shown aberrant lncRNA expression in neurological diseases [118,119]. Further, mounting studies have suggested potential roles for lncRNAs in immunity [120,121].

### 4.5. lncRNA Structure and Function

Similar to mRNAs, distinct mature ncRNAs can be obtained from primary non-protein coding RNA transcripts via alternative splicing in various differentiated cells, developmental stages or physiological states. It has been estimated that 95% of human primary transcripts of genes containing multiple exons are regulated by alternative splicing [122]. Alternative splicing produces transcript diversification [123]. Alternative splicing of pre-mRNAs generates circular RNA (circRNA) isoforms, ncRNAs with circular structures formed by covalent bonds without a 5′ terminal cap or a poly A tail [124]. In general, canonical splicing processes pre-mRNA sequentially in a 5′ to 3′ direction. The processing involves two transesterification reactions to form the intron lariat, followed by the orderly linkage of upstream and downstream exons [125]. However, in the models of the formation of circRNAs, the presence of a non-canonical transcription start determines that an orphan upstream 3′ exon splice site could be generated and then paired with a downstream 5′ exon splice site with introns being excised, which produces a circRNA with a circular structure [124]. Trans-splicing and exon skipping are two potential mechanisms by which circRNAs can be generated [126]. Alternative splicing produces many isoforms of the new discovered lncRNA ANRIL associated with different expression patterns and single nucleotide polymorphisms (SNPs). In general, introns are rapidly excised after transcription. However, more than 100 human introns have their 3′ tails degraded but retain their 2′,5′-phosphodiester bond at the splice site without being hydrolyzed. The reserved introns accumulate to form circular intronic lncRNAs (ciRNAs). At the 5′ and 3′ ends of ciRNAs, there are snoRNA structures that replace the 5′ cap and poly A tail and facilitate the accumulation of ciRNAs [127]. Existing evidence has shown that ciRNAs play cis-regulatory roles in the transcription of their parental genes through an interaction with the Pol II machinery [128]. The early discovered lncRNA Nuclear Enriched Abundant Transcript 1 (NEAT1) (MEN ε/β) has been shown to generate distinct isoforms (MEN ε and MEN β) by the alternative processing of the NEAT1 3′ end. MEN ε is characterized by poly A at its 3′ end, whereas, similar to the lncRNA (MALAT1), the 3′ end of MEN β consists of a triple helix structure [129]. Intriguingly, the structure of MEN β is more stable in various species, and the reason for this is currently under investigation [130].

It is currently accepted that the explanation for the various functions of lncRNAs lies in their multiple structures. Mounting evidence has revealed that some lncRNAs and circRNAs can serve as miRNA sponges and inhibit the binding of miRNAs to their target mRNAs to perform their functions [131]. Maternally expressed gene 3 (MEG3), which is highly expressed in the human pituitary, is an imprinted gene that can exist as 12 different transcriptional isoforms due to alternative splicing. All of the MEG3 isoforms have been recognized to inhibit tumor cell growth. The secondary structure motifs M1, M2 and M3 were observed in all of the MEG3 isoforms, and the M2 and M3 motifs have been shown to be closely involved in the activation of P53 and the inhibition of tumor cell growth [132]. However, some lncRNA isoforms perform opposing roles in biological processes. It is reported that the tumor suppressor gene PTEN is regulated by its pseudogene (PTENpg1) through the miRNA sponge action of PTENpg1. To further investigate this regulatory mechanism, two PTENpg1 antisense RNAs (asRNAs) were discovered to play opposing roles in the regulation of PTEN [133]. X-chromosome inactivation (XCI) is a common phenomenon in epigenetic processes. The lncRNA Xist (X-inactive specific transcript) is reported to act as a critical suppressor of X-chromosome inactivation (XCI) [134,135,136]. Several tandem repeat units composed of two stem-loop structures at the 5′ end of Xist have been shown to be essential for the initiation of XCI [51]. Circular ANRIL (cANRIL) is an ANRIL isoform whose circular structure is a by-product of pre-mRNA alternative splicing. Previous studies suggest that alterations of the structure and/or expression of ANRIL isoforms regulate the expression of INK4/ARF and are associated with atherosclerotic vascular disease (ASVD) [137]. MALAT1, also called nuclear-enriched transcript 2 (NEAT2), has been used as a prognostic marker for the occurrence and development of several types of tumors [138,139,140]. At the post-transcriptional level, the specific secondary structure at the 3′ end of the MALAT1 primary transcript can be recognized by RNase P and RNase Z, generating a triple helix structure that stabilizes MALAT1 and enables MALAT1 to perform its functions [129,141]. The ncRNA growth arrest-specific 5 (Gas5) is predicted to contain several specific hairpin structures and to be involved in starvation-induced cell survival and metabolic activities through the regulation of glucocorticoid receptor (GR) transcription [142].

## 5. Structural Prediction of ncRNAs

To elucidate the functions of lncRNAs and to further investigate the question of whether nucleotide sequences serve as functional units or simply linkers of different functional modules, it is necessary to study the structures of lncRNAs and the interaction between their structure and sequence. RNA possesses a unique ability to form complex secondary and tertiary folds [29]. It has been gradually recognized that the structural flexibility of RNA enables it to perform organizational, catalytic and regulatory functions [25,142,143]. It is now becoming feasible to obtain the functional annotation of transcriptomes based on RNA structure [28]. Traditional methods to investigate RNA structure include chemical probing [144], X-ray crystallography and NMR [145,146,147,148]. However, an increasing number of lncRNA molecules have been discovered. Due to the rapid degradation and difficult crystallization of RNA molecules, it is difficult to determine their stereo-chemical structure with these traditional approaches [28]. It is necessary to develop powerful computational methods to predict RNA structure. In this section, various structure prediction methods for noncoding RNAs are reviewed.

### 5.1. Prediction of ncRNA Secondary Structure

The folding process of the majority of RNA molecules represents a transition from secondary to tertiary structure [149]. Therefore, obtaining the RNA secondary fold is the first step in exploring the functions of ncRNAs [29]. In recent years, various methods have been proposed for predicting RNA secondary structure. These methods are based on two distinct ideas: multiple sequence alignments and the minimum free energy model [28].

#### 5.1.1. Multiple Sequence Alignments

Methods based on comparative sequence analysis rely on the fact that the structural conservation is greater than the sequence conservation of RNA [150,151]. Comparative sequence analysis compares several RNA sequences with similar secondary structures to search for conserved secondary structural units and predicts the secondary structure of an unknown RNA sequence [152].

##### Foldalign

Foldalign [30], simplified from Sankoff [153], utilizes a dynamic programming algorithm to find the highest scoring local alignment between a sequence and an alignment of other sequences or between two sequences [154]. The correlation coefficient [155] ranges from 0.8 to 0.9 between the verified database and the predicted structural alignments. Foldalign compares each sequence with every other sequence, and the numbers of the highest scoring alignments are saved. It can effectively perform on RNA sequences less than 300 nt. In addition, the time associated with this method is significantly reduced compared with the Sankoff version and other variants. However, the speed and efficiency of Foldalign require improvements [154]. The web server can be accessed at http://rth.dk/resources/foldalign/ [156].

##### Dynalign

Dynalign [157], which is based on a dynamic programming proposed by Sankoff, searches for a structure with low free energy common to two sequences without sequence identity by combining comparative sequence analysis and free energy minimization. Compared with free energy minimization alone, the average accuracy of this algorithm is improved from 47.8% to 86.4% for 5S rRNAs. It can predict a set of suboptimal secondary structures and create dot plots to read the information contained in suboptimal structures. Moreover, enzymatic cleavage data [158] and chemical modification probing experiments [159] can be applied to increase the prediction accuracy. However, it cannot predict pseudoknots, and the calculation is limited to sequences whose lengths are less than 400 nt [160].

##### Pfold

Pfold [161] is based on the KH-99 algorithm [162], which combined evolutionary information and a probabilistic structure model. Pfold can accommodate larger numbers of sequences, which can compensate for the limitations of the KH-99 algorithm. Due to its high computational speed and prediction accuracy, it is able to predict RNA secondary structure when long sequences and large numbers of homologous sequences need to be analyzed. With six sequences, an accuracy of 75% is attainable. In addition, many more sequences can be accommodated by Pfold, allowing for even higher accuracies [31]. However, there is still much room for this method to be improved, such as the introduction of a grammar to describe native-like RNA structures, stacking interactions and other models for base-pair evolution [161]. In addition, it cannot predict pseudoknots. Pfold is available through the web-based server www.daimi.au.dk/~compbio/pfold [163].

##### Alifold

The Alifold service [164,165], an extension of Zuker’s algorithm [166], uses modified dynamic programming algorithms combined with a covariance term to compute the consensus secondary structure of a set of aligned RNA sequences. It can predict minimum free energy structures and pair probabilities. The current limit for the length of the alignment is 3000 nt [165]. The advantages and limitations of Alifold are almost identical to those of RNAfold. This service can be accessed via the Vienna RNA web server at http://rna.tbi.univie.ac.at/cgi-bin/RNAalifold.cgi [167].

##### MARNA

MARNA [168], a non-probabilistic approach [169], performs pairwise alignments considering both the primary and secondary structures. It folds sequences using the minimum free energy and then provides structural alignment among a set of homologous sequences. When the conservative sequence regions are invisible, MARNA is an appropriate option to predict RNA secondary structure. Users can designate individual parameters that can set the weight for either sequence or structural properties. However, the total length of sequences should not be longer than 10,000 nt. MARNA can be used online on the following webpage: http://rna.informatik.uni-freiburg.de/MARNA/Input.jsp [170].

A large number of studies and experiments have demonstrated that comparative sequence analysis processes higher prediction probabilities when the RNA sequence templates have high similarity [171]. However, because comparative sequence analysis depends on the prior knowledge of sequences, this model is unfit for single RNA sequences or sequences from considerably different sources [152]. In addition, comparative sequence analysis is time- and internal storage-consuming, which limits its application for predicting longer RNA sequences [28].

#### 5.1.2. Minimum Free Energy Model

When no prior knowledge is available and only a single sequence is offered, an accurate and popular method is to search the minimum free energy model through thermodynamic computation [172]. This model utilizes efficient dynamic programming algorithms to search for a secondary structure with the minimum free energy [166]. However, true RNA secondary structure may not be the structure with the minimum free energy. Zuker *et al.* [173] developed the concept of suboptimum structures. All suboptimum structures must be further identified by biology researchers.

##### Mfold

Mfold [32] divides RNA secondary structures motifs into the stem area, bulge loop, internal loop and hairpin loop. Different computational methods are used to calculate the free energy of different motifs. Then, the motifs are assembled through dynamic programming algorithms, and the secondary structure with the minimum free energy can be obtained. Using this method, prior knowledge can be specified before the prediction; the structure of circular RNA sequences is predictable, the maximum for internal or bulge loops can be set, and the maximum distance between paired bases can be artificially determined. Many studies have proposed that RNA secondary structure affects splicing activity [174]. Yun Yang *et al.* [175] discovered that the inherent intronic elements are underlying mechanisms for the pre-mRNA splicing process. These elements have been found conserved at the RNA secondary structural level. In their studies, the Mfold program was used to predict intronic pairings. However, Mfold can only predict the secondary structure of single stranded RNA. The portal for the Mfold webserver is http://unafold.rna.albany.edu/?q=mfold [176].

##### RNAfold

RNAfold [33], which is based on dynamic programming algorithms and computations of the equilibrium partition functions and base pairing probabilities, uses the minimum free energy model and multiple sequence alignments when given single stranded RNA sequences and several stranded RNA sequences, respectively. RNAfold is a reliable option regardless of whether the base pairing of G and U is acceptable or not. Moreover, the sequences can contain incorrect characters. Furthermore, the program can predict single stranded and several stranded RNAs. Humann *et al.* [177] discovered differentially expressed lncRNAs in the larval ovaries of honeybee caste by using the RNAfold program and other biological technologies. They named the newly discovered lncRNAs lncov1 and lncov2. The secondary structures of both RNAs consist of several consensus hairpin motifs lacking coding potential. However, it is worth noting that the length of the sequence should not be more than 300 nt. When predicting several stranded RNAs, the program can only produce the consensus structure as opposed to the secondary structure of each sequence. In addition, the total length of the sequences cannot exceed 10K nt when predicting the consensus structure. The portal for the RNAfold web server is http://rna.tbi.univie.ac.at/cgi-bin/RNAfold.cgi [178].

##### RNAshapes

RNAshapes [34], based on the abstract shapes approach [179], is a new method that combines three RNA analysis tools: the analysis of shape representatives, the consensus shapes approach and the calculation of shape probabilities. Compared with other current RNA folding algorithms, RNAshapes only describes classes of structures from concrete secondary structures. These structures fall into different shape categories. Within a shape class, every representative is the secondary structure with the minimum free energy. Using this package, the single-stranded RNA, the sequence files and the multi-sequence files are all predictable. For a given threshold value, the number of shapes is less than the number of structures, and the native structures are among the shape representatives. Therefore, users can avoid researching redundant suboptimal structures [179]. However, because the folding kinetics are not considered, the minimum free energy prediction may be incorrect. RNAshapes is freely available at http://bibiserv.techfak.uni-bielefeld.de/rnashapes [180].

##### RNAstructure

RNAstructure [35] utilizes the most recent set of thermodynamic parameters to implement the nearest neighbor parameters as determined by the Tuner group [181,182] based on dynamic programming algorithms and Sankoff, which allow sequence alignment and structure prediction to proceed simultaneously. The user interface is friendly and powerful. Its “Max % Energy Difference” and “Max Number of Structures” can be modified to limit the number of suboptimal structures predicted. Moreover, experimental data can be added to constrain the structures. Furthermore, it can predict both single stranded RNA and a structure common to two sequences. This method has been widely used in research. Ding *et al.* [183] compared the structural features of mRNAs *in vivo* with predicted structures (determined by RNAstructure) *in silico* and revealed that mRNAs related to stress responses have structural features, such as longer maximal loop length and more single strandedness, that allow for easy conformational changes under various environmental conditions. SPRY4-IT1, the lncRNA that regulates invasion and apoptosis, was predicted to contain long hairpin motifs (by RNAstructure), suggesting that SPRY4-IT1 may function as an RNA molecule [184]. The package is available for downloading at http://rna.urmc.rochester.edu/RNAstructure.html [185].

The information regarding the methods described above is summarized in Table 1. Apart from the mainstream methods mentioned above, Sfold [186,187], Contrafold [188], and MPGAfold [187] are also available to solve problems when predicting RNA secondary structure. Although there has been remarkable development in the methods to predict RNA secondary structures, the methods based on the free energy parameters proposed by Zuker *et al.* [32,173] still represent the mainstream.

**Table 1:** Comparison of the various major methods to predict RNA secondary structure.

Method	Principle	Advantages	Limitations
Foldalign [30]	Sankoff, dynamic programming algorithm	time complexity decreased	length of sequence shorter than 300 nt; low speed and efficiency
Dynalign [158]	Sankoff, dynamic programming algorithm	suboptimal secondary structures accessible; constrained information added	Pseudoknots not predictable
MARNA [169]	folding sequences using minimum free energy; proceedings structural alignment	individual parameters freely set	total length of sequences shorter than 10,000 nt
Mfold [32]	Zuker’s dynamic programming algorithm based on minimum free energy model	priori knowledge specified; structure of circular RNA sequence predictable; some values related to structure artificially made	only structure of single stranded RNA predictable
Alifold/RNAfold [33,165]	minimum free energy model and multiple sequence alignment	containing incorrect characters; single stranded RNA and several stranded RNAs predictable; base pairing of G and U acceptable	when predicting several stranded RNAs, only producing consensus structure instead of the secondary structure of each sequence; when predicting single sequence, its length requirement is less than 300 nt; total length of sequence not to exceed 10K nt when predicting consensus structure
RNAshapes [34]	abstract shapes approach	single stranded RNA, sequence files and multi-sequence files predictable; redundant suboptimal structures avoided	does not consider folding kinetics; minimum free energy prediction may be incorrect
RNAstructure [35]	dynamic programming algorithm and Sankoff	number of suboptimal structures limited; structures constrained by experimental data	only AGCU predictable

### 5.2. Prediction of ncRNA Tertiary Structure

The formation of specific tertiary structures is essential for the functioning of noncoding RNAs in many biological processes [189]. RNAs can alter their tertiary structure under different conditions, enabling them to interact with other RNAs, ligands, proteins or themselves [28]. In this section, methods to predict the tertiary structure of ncRNAs are reviewed.

#### FARNA

FARNA [190], derived from the Rosetta methods of protein tertiary structure prediction [191], utilizes coarse-grained models as dummy atoms to replace the center of each base and seek RNA tertiary structure with the minimum free energy. The prediction accuracy of the main chains can reach a 4 Å root-mean-square-deviation (RMSD) [192] for short RNA sequences with a length less than 30 nt. The prediction accuracy of this method can be further improved by combining it with experimentally determined secondary structure information [193]. In recent years, Baker *et al.* [194] have introduced all-atom items to FARNA, which has allowed FARNA to become an all-atom structure prediction method. FARNA is characterized by a better computational efficiency in comparison with numerous sampling strategies. However, FARNA can only predict the tertiary structure of small RNA molecules (<40 nt). Challenges remain in accommodating RNA molecules of longer lengths or with complex topological structures.

#### NAST

NAST (The Nucleic Acid Simulation Tool) [195], based on coarse-grained models, uses knowledge-based energy functions to automatically predict RNA tertiary structure. NAST requires secondary and tertiary contact information for target RNA molecules to direct folding. It has a mean RMSD of 8.0 ± 0.3 and 16.3 ± 1.0 Å for the yeast phenylalanine tRNA and the P4–P6 domain of the Tetrahymena thermophila group I intron, respectively. Plausible RNA structures can be created with empirical RNA geometric distributions, a relatively high modeling speed can be achieved by using single-point-per-base models, and the capacity to constrain and filter models with experimental data improves the prediction accuracy of NAST. Due to computational complexity, modeling large RNA molecules remains difficult. The software package is freely available at https://simtk.org/home/nast [196].

#### iFoldRNA

iFoldRNA [37] uses discrete molecular dynamics (DMD) to rapidly explore RNA tertiary conformation [36,197]. Compared with traditional dynamic molecule simulations, the rapid conformation sampling ability of DMD contributes to its rapid structure prediction [198]. Low RMSDs (2–3 Å) are observed in the predictions of iFoldRNA. iFoldRNA can predict the tertiary structure of small RNA molecules (<50 nt) with simple topological structure. When predicting larger RNA molecules (>50 nt), a longer time is required to sample conformational space, which exponentially increases. Recently, parameters including base pairing, base-stacking, and hydrophobic interactions obtained from experiments have been integrated into iFoldRNA to constrain the structures of larger RNA molecules [199].

#### BARNACLE

BARNACLE [200], a probabilistic model of RNA structure, provides sampling of RNA conformations in continuous space. The current state of prediction methods such as FARNA are primarily based on combining short fragments obtained from experiments to construct reasonable native-like tertiary structures. However, there are some computational sampling problems associated with these methods. It is possible for BARNACLE to efficiently sample 3D conformations of RNA on a short length scale. BARNACLE can accurately predict RNA tertiary structure when the length of the RNA sequence is less than 50 nt (10 Å RMSD). Nevertheless, structure sampling becomes difficult due to too many degrees of freedom with longer RNA molecules or with those that harbor complicated topological structures. Moreover, the sequence and evolutionary information of BARNACLE needs to be extended.

#### CG Model

The CG model [201] models RNA structures with molecular dynamics based on a new statistical coarse-grained potential. The statistical analysis of 688 RNA experimental structures has been applied to parameterize the CG potential [202]. The computational efficiency is greater than that of the all-atom model because of the reduction in the number of angles, bonds and torsion calculations. Fifteen RNA molecules with a length of 12 to 27 nt have been tested through molecule dynamics simulation, this shows that 75% of RNA molecules can be led to native-like structures with at least one out of multiple pathways using the simulated annealing method. If secondary or tertiary structure interaction information is provided, all of the RNA molecules will successfully be folded into structures with an RMSD less than 6.5 Å. Similar to other methods, this method is restricted to predicting small RNA molecules with simple topological structures.

#### RNA2D3D

RNA2D3D [203], different from other structure prediction methods, is based on unpaired bases derived from Assisted Model Building with Energy Refinement (AMBER) [204] and canonical base-pairings of the A-form helix to model RNA tertiary structure. However, overlapping atoms, covalent bond disassociation and other structural problems that exist in the RNA tertiary structure are automatically generated by RNA2D3D. Therefore, further optimization is necessary to obtain a reasonable RNA tertiary structure. After the adjustment and optimization of RNA2D3D, the pseudoknot structure of the telomerase RNA, with a length of 48 nt, has been successfully built by Shapiro *et al.*, and the RMSD reached 7 Å [205,206,207].

#### Vfold Model

The Vfold model [208] is a physics-based method for predicting larger and more complex RNA molecules from nucleotide sequences. This method uses a multi-scaling strategy in which secondary and tertiary structures are obtained in a serial fashion. Compared with other methods, the Vfold model can predict larger RNA molecules, for example the 122-nt 5S rRNA domain (RMSD 7.4 Å). The most significant advantage of the Vfold model is its statistical mechanical calculations for the conformational entropy of RNA tertiary structures. In addition, the model can be used to predict all low-lying tertiary structures in the energy landscape. However, this method does not consider the sequence-dependent tertiary contacts, such as general loop-loop and loop-helix interactions, in loop-free energy minimization.

#### RSIM

RSIM [36], a fully automated application, is an improved approach to predict RNA tertiary structure using the fragment assembly method based on RNA secondary structure constraints. It overcomes the pitfalls of FARNA, such as the reduction of the size of the sampled conformational space and the reasonable base-pairing constraint using the fragment assembly method. Monte Carlo simulations, a statistical potential and a diverse fragment library are further used to refine the tertiary structures obtained by RSIM. During the refinement, the stimulation paths can be tracked. RSIM can accommodate RNA molecules with a length over 40 nt (RMSD 4.8 Å). However, RSIM cannot automatically predict the tertiary structure of RNA molecules with pseudoknot structures. RSIM is available at http://www.github.com/ jpbida/rsim [209].

#### 3dRNA

3dRNA [38], based on RNA sequence and secondary structural information, is a method for the rapid and automated building of RNA tertiary structure. It is a hierarchical approach to the construction of RNA tertiary structure [210]. Compared with other methods, 3dRNA can obtain RNA tertiary structural templates from different RNA families. It is found that the conformations of the backbone of RNA structural templates of the same sequence are similar to each other. These changes contribute to a high average prediction accuracy of 3.97 Å RMSD. 3dRNA is not limited to predicting the tertiary structures of small RNA molecules or those with simple topology. For RNA molecules of a large size and complex topology, the predicted tertiary structures have an average RMSD of 5.7 Å. The research conducted in Qian’s lab in Northwestern Polytechnical University has predicted the tertiary structures of 5 lncRNAs with 3dRNA and uncovered important roles for these lncRNAs in bone formation when MACF1 (Microtubule actin cross-linking factor l) is down-regulated (data not shown). The package is available at http://biophy.hust.edu.cn/3dRNA/3dRNA-1.0.html [211].

The methods mentioned above are widely used to predict RNA tertiary structure. Furthermore, MC-Fold/MC-Sym [212], based on the nucleotide cyclic motif (NCM), is a first-order object to represent nucleotide relationships in structured RNAs. ASSEMBL [213] is an interactive graphical tool based on human-computer interactions to analyze and build 2D and 3D RNA models. In general, the prediction accuracy of RNA tertiary structure will be largely improved by the addition of structural information, such as RNA secondary structure, distance, rotation angle, dihedral angle and other tertiary structural information [214]. However, Liang and Schlick [215,216] accessed these existing RNA tertiary structure prediction methods and found that they are restricted to analyzing short (<50 nt) or topologically simple molecules with RMSD less than 6 Å. When predicting larger (50 to 130 nt) or more topologically complex RNA molecules, the tertiary structure can be obtained with a mean RMSD of 20 Å. Moreover, the existing prediction methods for RNA tertiary structure require human-computer interactions for further adjustment to optimize the obtained RNA tertiary structure. Therefore, the proposal of 3dRNA is a significant step forward in the prediction of RNA tertiary structure. The various methods for predicting RNA tertiary structure are summarized in Table 2.

**Table 2:** Various methods for predicting RNA tertiary structure.

Method	Principles	Advantages	Limitations
FARNA [36]	coarse-grained models, minimum free energy	better computational efficiency	small RNA molecules (<40 nt)
NAST [195]	coarse-grained models, knowledge-based energy function	relatively high modeling speed; constraint models	computational complexity
iFoldRNA [37]	discrete molecular dynamics	rapid conformational sampling ability	small RNA molecules (<50 nt)
BARNACLE [201]	probabilistic model, sampling of RNA conformations in continuous space	efficient sampling of 3D RNA conformations on a short length scale	small RNA molecules (<50 nt); sample difficulty
CG Model [202]	molecular dynamics based on a new statistical coarse-grained potential	high computational efficiency	small RNA molecules or those with simple topology
RNA2D3D [204]	base-pairing structure of RNA molecules	can predict pseudoknots	obtaining reasonable RNA tertiary structure to be solved
Vfold Model [209]	physics-based method	statistical mechanical calculations for the conformational entropy of RNA tertiary structures	does not consider the sequence-dependent tertiary contacts
RSIM [36]	fragment assembly	the reduction in the size of conformational space sampled; reasonable base-pairing constraints	RNA molecules with pseudoknot structures not automatically predictable
3dRNA [38]	hierarchical approach to construct RNA tertiary structure	highest prediction accuracy	ability to model larger RNA molecules or those with complex topology

## 6. Conclusions

With an increasing number of studies focused on lncRNAs, an increased understanding of lncRNAs has been achieved. lncRNAs play biological roles in organisms, and their dysregulation is strongly linked to the occurrence and development of various diseases [217]. However, the in-depth knowledge of the function of lncRNAs is a developing but difficult field due to the diversity and complexity of the mechanisms underlying lncRNAs. As RNA function is closely associated with its structure [24], analyzing RNA structure provides a new approach to the study of lncRNAs [28]. Before major progress in the determination of ncRNA structure using physical methods is achieved, the structural prediction of ncRNAs will be a hotly debated issue. At present, the prediction of pseudoknots is very difficult [218]. Our knowledge of thermodynamics [182,219] and algorithms to model RNA molecules undergoing conformational changes [28] is incomplete. These represent problems that need to be addressed for the secondary structure prediction of ncRNAs. Moreover, with the ongoing improvements in the accuracy of ncRNA structural prediction, it is possible to reliably predict the tertiary structure of small RNA molecules; however, predicting the structure of large RNA molecules or those with complex topological structures [38] remains challenging. Moreover, tackling the structure of non-canonical base pairings in the prediction of RNA tertiary structure remains a difficult problem [38]. Furthermore, to elucidate the complicated mechanisms of actions of lncRNAs, the use of experimental data as constraint information is inevitable. It is expected that issues occurring in the structural prediction of lncRNAs will be addressed in the future and that additional techniques will be applied to studies of lncRNA function, which will allow the further analysis of their functions, molecular regulation and pathological mechanisms in diseases. In the future, lncRNAs may serve as drug targets and provide new opportunities for the treatment of diseases.

## Abbreviations

lncRNAs: long noncoding RNAs; ncRNAs: noncoding RNAs; NATs: natural antisense transcripts; PRC2: Polycomb Repressive Complex 2; NAST: The Nucleic Acid Simulation Tool; AMBER: Assisted Model Building with Energy Refinement; MACF1: Microtubule actin cross-linking factor l.
